# Depression and bipolar disorder subtypes differ in their genetic correlations with biological rhythms

**DOI:** 10.1038/s41598-022-19720-5

**Published:** 2022-09-21

**Authors:** Lea Sirignano, Fabian Streit, Josef Frank, Lea Zillich, Stephanie H. Witt, Marcella Rietschel, Jerome C. Foo

**Affiliations:** Department of Genetic Epidemiology in Psychiatry, Medical Faculty Mannheim, Central Institute of Mental Health, University of Heidelberg, J5, 68159 Mannheim, Germany

**Keywords:** Genetics, Genomic analysis, Psychiatric disorders, Bipolar disorder, Depression

## Abstract

Major Depression and Bipolar Disorder Type I (BIP-I) and Type II (BIP-II), are characterized by depressed, manic, and hypomanic episodes in which specific changes of physical activity, circadian rhythm, and sleep are observed. It is known that genetic factors contribute to variation in mood disorders and biological rhythms, but unclear to what extent there is an overlap between their underlying genetics. In the present study, data from genome-wide association studies were used to examine the genetic relationship between mood disorders and biological rhythms. We tested the genetic correlation of depression, BIP-I, and BIP-II with physical activity (overall physical activity, moderate activity, sedentary behaviour), circadian rhythm (relative amplitude), and sleep features (sleep duration, daytime sleepiness). Genetic correlations of depression, BIP-I, and BIP-II with biological rhythms were compared to discover commonalities and differences. A gene-based analysis tested for associations of single genes and common circadian genes with mood disorders. Depression was negatively correlated with overall physical activity and positively with sedentary behaviour, while BIP-I showed associations in the opposite direction. Depression and BIP-II had negative correlations with relative amplitude. All mood disorders were positively correlated with daytime sleepiness. Overall, we observed both genetic commonalities and differences across mood disorders in their relationships with biological rhythms: depression and BIP-I differed the most, while BIP-II was in an intermediate position. Gene-based analysis suggested potential targets for further investigation. The present results suggest shared genetic underpinnings for the clinically observed associations between mood disorders and biological rhythms. Research considering possible joint mechanisms may offer avenues for improving disease detection and treatment.

## Introduction

Major Depression (MD) and Bipolar disorder (BIP) are common (lifetime prevalence MD: 20.6%, BIP-I: 0.6%, and BIP-II: 0.4%), often chronic disorders that can cause harm to those affected and the people close to them^1,2^. MD is characterized by depressive episodes, while BIP is characterized by manic (BIP-I) or hypomanic (BIP-II) episodes, generally alternating with depressive episodes^3^.

Changes in physical activity, circadian rhythm, and sleep, hereafter referred to collectively as “biological rhythms”, are often observed in mood disorders. Biological rhythms is a term used to summarize a series of body functions determined by the internal circadian clock (e.g., activity, sleep, body temperature, etc.)^4^. Different patterns of biological rhythms are generally observed during different mood episodes (depressed, manic, hypomanic). The cardinal symptoms of a depressive episode are a persistent feeling of sadness and/or loss of interest in almost all activities. Further symptoms include feelings of worthlessness or guilt, suicidality, poor concentration, loss of weight, tiredness and loss of energy, disturbances of sleep, and psychomotor alterations. Manic episodes and hypomanic episodes (which are less intense) are characterized by an ongoing inappropriate elevated or irritable mood. Further symptoms include an excessive sense of self-worth or grandiosity, being more talkative and more easily distracted, reduced need for sleep, and markedly increased energy and activity levels and/or psychomotor agitation^3^.

There is an increasing number of studies investigating such alterations of biological rhythms in mood disorders with objective assessments. Actigraphy studies observe lower activity levels in individuals with MD compared to healthy controls^5,6^. The same is found for individuals with BIP^7,8^. When comparing the different mood states (depressed, manic, hypomanic) within BIP, significantly higher levels of activity during manic and hypomanic phases are observed than in depressed phases^9,10^. However, actigraphy studies evaluate not only overall physical activity levels but investigate various other types of activity such as ‘sedentary behaviour’ or ‘moderate activity’ (for details, see Materials and Methods)^11^.

Circadian mechanisms govern many biological processes and their importance is being increasingly recognized in the etiology, diagnosis, and treatment of mood disorders^12^. A parameter commonly used as a proxy for circadian rhythms of body movement is relative amplitude; it is often used in actigraphy studies, where based on movement acceleration data, the average activity for the least active continuous 5 h of a day is subtracted from the average for the most active continuous 10 h; this is divided by their sum^13^. High relative amplitude values are often observed in healthy individuals reporting to be more active during the day and less active during the night, while patients with mood disorders usually describe the opposite pattern, reflected in low relative amplitude values^14,15^. Relative amplitude is a rest-activity parameter which can be used to describe the circadian rhythm/disruption of activity, but does not represent the circadian rhythms of different physiological body functions. Lower relative amplitudes are found in both depression and BIP compared to healthy individuals^13,16^.

Sleep is a recurring biological process holding many functions of great importance for the human body (e.g., restoration and memory functioning)^17^. Sleep problems, insufficient sleep, irregularity of sleep–wake cycles, and differences in sleep duration, are well described in MD and BIP^18,19^, as is daytime sleepiness, which is also reported to be increased in patients suffering from MD and BIP^20,21^.

Both mood disorders and biological rhythms have a genetic basis with heritability estimates for MD at 40% and BIP at 80%; for biological rhythms, those estimates are heterogeneous, ranging from small (< 30%) to high (78%) for physical activity, from 40 to 54% for chronotype, and 31–49% for normal sleep due to factors such as variation in age and study design^22–24^. It is uncertain whether changes in biological rhythms typically observed are a mere consequence of a given mood episode (e.g., a patient who is depressed and has lost interest would move less, showing decreased activity) or due to common etiology. This raises the question of whether genes increasing the liability for MD, BIP-I, and BIP-II overlap with genes predisposing individuals to altered biological rhythms and if so, whether this overlap differs between the different mood disorder subtypes.

The few formal genetic studies which have addressed these questions so far suggest a possible shared etiology. Twin studies observed negative genetic correlations of depressive symptoms with physical activity (n = 5952 twins, n = 756 twins)^25,26^ as well as with sleep duration (n = 894 twins)^27^. In a family study of 26 high-density bipolar families, analyses of actigraphy data revealed 13 trait-like associations with BIP-I, demonstrating lower activity levels than in their non-BIP-I relatives^28^.

Genetic associations between mood disorders and circadian rhythms have largely been studied in circadian-related clock genes (e.g., ARNTL, CLOCK, CRY, PER, etc.)^29,30^, given the many aspects of physiological processes under the control of the central circadian clock (for reviews, see^31–33^). Advances in molecular methods now offer the possibility to investigate such associations not only on a single-variant level but also on the gene- and genome-wide level. Increases in sample size, power, and number of genome-wide association studies (GWASs) of many disorders and traits have enabled *in silico* testing for genetic overlap of various phenotypes on a large scale^34^. In previous studies using such GWAS data to examine the overlap between mood disorders and altered biological rhythms, positive associations have been observed between depressive symptoms and daytime sleepiness^35^ and negative correlations between MD and physical activity^36^. In contrast, for BIP (subtypes unspecified), positive associations have been found with physical activity, but none with daytime sleepiness^35,36^.

The present study aims to explore whether depression, BIP-I, and BIP-II share an overall genetic etiology with biological rhythms using summary statistics from the most current respective GWASs^35,37–40^ independent of the current mood state. Differences in genetic correlations of depression, BIP-I, and BIP-II with biological rhythms are examined to identify commonalities as well as differences between the disorders and their relationship with biological rhythms. To gain deeper insight, associations of mood disorders and biological rhythms with single genes, particularly with known circadian genes, and the overlap of genes between mood disorders and biological rhythms, are explored.

## Materials and methods

### GWAS samples

Table 1 gives an overview of the GWAS summary statistics and cohorts used in this analysis. Summary statistics comprise aggregate *p*-values and association data for every single nucleotide polymorphism (SNP) analysed in a GWAS^34^.

**Table 1:** Overview of GWAS summary statistics analyzed.

Phenotype	Reference	Sample
Depression	Howard et al.^37^	170,756 Depression cases (excluding 23andMe)
BIP-I BIP-II	Mullins et al.^38^	25,060 BIP-I cases and 6,781 BIP-II cases
Overall physical activity Moderate activity Sedentary behaviour Sleep duration	Doherty et al.^39^	91,105 participants UK Biobank cohort
Relative amplitude	Ferguson et al.^40^	71,500 participants UK Biobank cohort
Daytime sleepiness	Wang et al.^35^	452,071 participants UK Biobank cohort

For depression, the latest publicly available summary statistics were used as described in^37^, the meta-analysis comprised 33 cohorts (excluding 23andme cohort), resulting in summary statistics of 170,756 depression cases and 329,443 controls. Summary statistics for BIP-I and BIP-II were derived from the latest Psychiatric Genomics Consortium GWAS on BIP consisting of 57 cohorts with a total number of 41,917 BIP cases and 371,549 controls, including 25,060 BIP-I, 6,781 BIP-II cases^38^.

Biological rhythms summary data were obtained from studies carried out in the UK Biobank (UKB) cohort. The UKB is a national cohort consisting of over 500,000 participants (40–69 years old) with genetic and deep phenotype data^41^. Biological rhythms were assessed in a subset of the UKB cohort including 103,720 individuals who wore accelerometers for 7 days. Based on this accelerometer data the UKB accelerometer expert working group extracted or classified several physical activity parameters: overall physical activity (measuring the mean activity over the whole assessment), moderate activity (activities requiring higher levels of energy such as exercising), sedentary behaviour (activities requiring low energy consumption like sitting or lying), relative amplitude (for circadian rhythm), and sleep duration (the time spent in behaviour classified as sleep)^11^. Daytime sleepiness was assessed subjectively in the whole UKB sample with a self-reported question, asking participants if they were likely to doze off or fall asleep during the day unintentionally (rating on a 4-point scale)^35^. The current analysis used summary data from GWASs based on these biological rhythm parameters were made available by the UKB.

All original studies and used datasets are included in the **Data Availability** section, where detailed information on cohorts and datasets can be found.

### Genetic correlation analysis

Genetic correlations of depression, BIP-I, and BIP-II, with overall physical activity, moderate activity, sedentary behaviour, sleep duration, relative amplitude, and daytime sleepiness were calculated using the bivariate genetic correlation method in Linkage Disequilibrium Score Regression (LDSC) software (https://github.com/bulik/ldsc). LDSC is a regression-based analysis tool that can estimate the genetic correlation between two traits using GWAS summary statistics^42^. We used the Linkage Disequilibrium (LD) Scores of the 1,000 Genomes Project for use with European samples. Default settings were applied for the filtering process: information metric (INFO score) > 0.9, indicating high quality of imputation, Minor Allele Frequency (MAF) > 0.01, and removing out of bounds summary statistic *p*-values. Indels, ambiguous, duplicated SNPs, and SNPs having no match in the LD Score reference panel were removed. SNP heritability analyses using LDSC revealed a Z-score of > 4 for all used GWAS summary statistics (see Tables S1.1–S1.3) indicating good interpretability and power for genetic correlation analyses, as recommended by the authors of LDSC^43^. For each mood disorder (depression, BIP-I, and BIP-II) genetic correlations with the six biological rhythm traits were estimated for which Bonferroni correction (*p* < 0.05/6 = 0.0083) was applied.

### Analysis of differences in correlations

To compare the above calculated correlations between depression, BIP-I, and BIP-II, we employed the block jackknife extension of LDSC^42^, as previously described in^44^. A z-test was conducted using the LDSC extension computing a z-value (z), standard error (SE), and *p*-value for each comparison. The following comparisons were analysed: depression vs. BIP-I, depression vs. BIP-II, and BIP-I versus BIP-II in their correlations with biological rhythm traits. For each comparison, six tests were conducted and Bonferroni correction (*p* < 0.05/6 = 0.0083) for the number of biological rhythm traits was applied.

### Gene association analysis and targeted examination of circadian genes

A genome-wide gene-based analysis for mood disorders and biological rhythms was conducted using the Multi-marker Analysis of GenoMic Annotation (MAGMA)^45^. MAGMA is a computational tool that allows gene-based and geneset analysis of GWAS data and is widely used to detect genes and pathways associated with phenotypes and diseases. The gene-based analysis is based on a multiple regression model, using data of all single variants annotated to a gene, deriving one p-value per gene to determine if the genetic variation in this gene is associated with the phenotype (for further details see^45^).

Default settings were used. Lists of genes were reduced to the number of genes available in all mood disorders and biological rhythms summary statistics (n = 17,861 genes). Bonferroni correction was applied for all tested genes (*p* < 0.05/17,861 = 2.80 × 10^–6^) to determine genes significantly associated with mood disorders and biological rhythms.

The overlap of genes associated with each of the mood disorders and biological rhythms was then examined based on filtered gene sets with a suggestive threshold of *p* < 1 × 10^–5^.

Next, utilizing MAGMA results, we took a targeted look at associations of mood disorders with common circadian genes *(ARNTL, CLOCK, CRY1, CRY2, DBP, NPAS2, NR1D1, NR1D2, PER1, PER2, PER3, RORA, RORB, RORC)*^32^ applying a Bonferroni corrected significance threshold, correcting for the number of tested circadian genes (*p* < 0.05/14 = 0.004).

## Results

**Figure 1:**
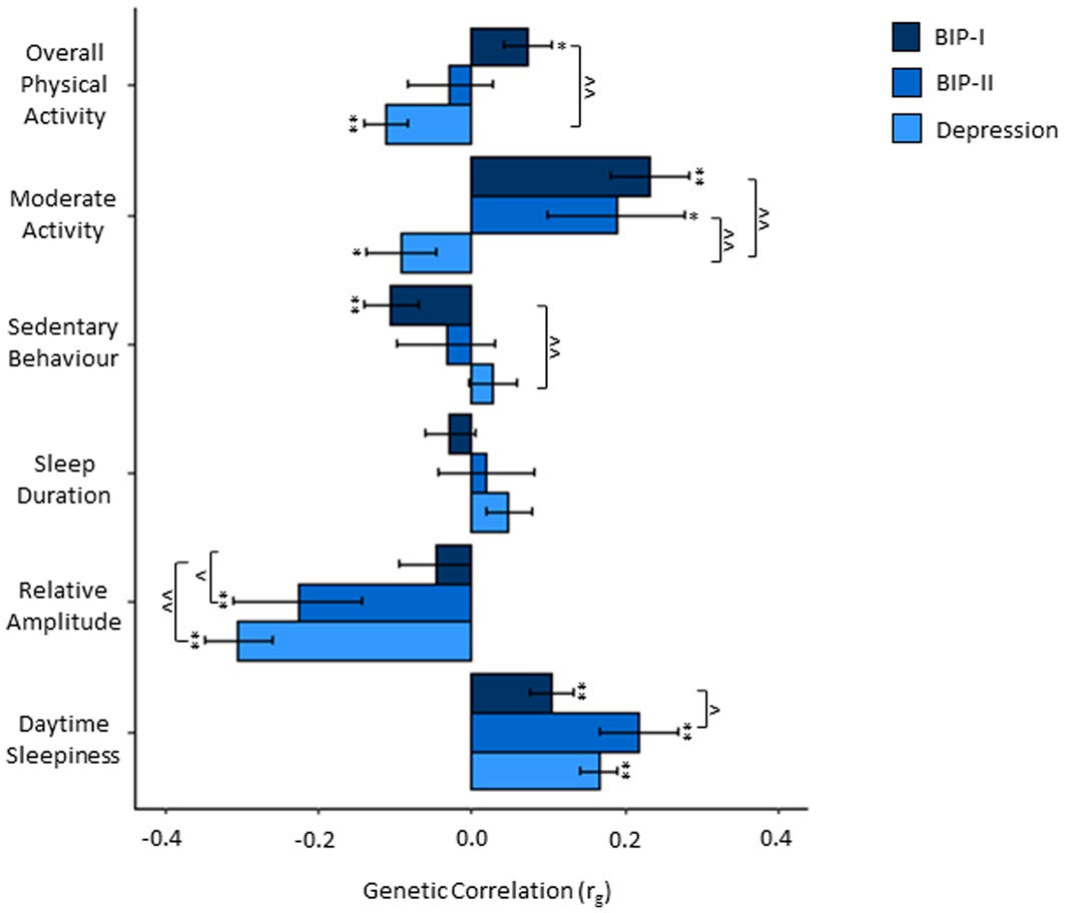
Genetic Correlations of Depression, BIP-I, BIP-II with Biological Rhythms. r_g_ = genetic correlation coefficient; error bars indicate standard error limits of r_g_; **p_adj_ < 0.05, *p < 0.05; for comparison of correlations: ^^*p*_adj_ < 0.05, ^*p* < 0.05.

Genetic correlations of depression, BIP-I, and BIP-II with biological rhythm traits and the differences between the mood disorders in their correlations are presented in Fig. 1. Bonferroni corrected p-values are indicated in Fig. 1 and reported in Tables S1.1–S2.3. Bonferroni corrected p-values from the gene-based analysis of all 17,861 tested genes and common circadian genes are shown in Supplementary Tables S3.1–S4.9.

### Genetic correlations of depression with biological rhythms

Significant negative correlations of depression with overall physical activity (r_g_ = − 0.11, *p* = 0.0001), moderate activity (r_g_ = − 0.09, *p* = 0.04) and with relative amplitude (r_g_ = − 0.30, *p* = 4.40 × 10^–12^) were found. A significant positive correlation of depression with daytime sleepiness (r_g_ = 0.17, *p* = 3.17 × 10^–11^) was observed. Depression was not significantly correlated with sedentary behaviour (r_g_ = 0.03, *p* = 0.40), and sleep duration (r_g_ = 0.05, *p* = 0.11) (see Table S1.1).

### Genetic correlations of BIP-I with biological rhythms

BIP-I was positively correlated with overall physical activity (r_g_ = 0.07, *p* = 0.02), moderate activity (r_g_ = 0.23, *p* = 4.98 × 10^–6^), and daytime sleepiness (r_g_ = 0.11, *p* = 0.0002) and negatively correlated with sedentary behaviour (r_g_ = − 0.11, *p* = 0.003). BIP-I was not significantly correlated with sleep duration (r_g_ = − 0.03, *p* = 0.39) and relative amplitude (r_g_ = − 0.05, *p* = 0.31) (see Table S1.2).

### Genetic correlations of BIP-II with biological rhythms

BIP-II was positively correlated with moderate activity (r_g_ = 0.19, *p* = 0.04) and daytime sleepiness (r_g_ = 0.22, *p* = 2.36 × 10^–5^), and negatively correlated with relative amplitude (r_g_ = − 0.23, *p* = 0.006). BIP-II was not significantly correlated with overall physical activity (r_g_ = − 0.03, *p* = 0.62), sedentary behaviour (r_g_ = − 0.03, *p* = 0.61), and sleep duration (r_g_ = 0.02, *p* = 0.77) (see Table S1.3).

### Differences between correlations

Depression and BIP-I differed significantly in their correlations with overall physical activity (z = − 4.33, *p* = 1.50 × 10^–5^), moderate activity (z = − 4.80, *p* = 1.57 × 10^–6^), sedentary behaviour (z = 2.95, *p* = 0.003), and relative amplitude (z = − 4.15, *p* = 3.36 × 10^–5^). Depression and BIP-I did not differ significantly in their correlations with sleep duration and daytime sleepiness (see Table S2.1).

Depression and BIP-II differed significantly in their genetic correlations with moderate activity (z = − 2.70, *p* = 0.007). The correlations of depression and BIP-II with overall physical activity, sedentary behaviour, sleep duration, relative amplitude, and daytime sleepiness did not differ significantly (see Table S2.2).

BIP-I and BIP-II differed significantly in their correlation with relative amplitude (z = 2.17, *p* = 0.03) and daytime sleepiness (z = − 2.31, *p* = 0.02). Comparing correlations of BIP-I and BIP-II with respect to overall physical activity, moderate activity, sedentary behaviour, sleep duration yielded no significant differences (see Table S2.3).

### Gene association analysis and examination of circadian genes

100 genes were significantly (p_adj_ < 0.05) associated with depression; the top-ranked were *HIST1H2BN*, *HIST1H3J*, and *SORCS3* (see Table S3.1). BIP-I was signficantly associated with 122 genes, the top-ranked were *CACNA1C*, *MAD1L1*, and *PLEC* (see Table S3.2). *SLIT3* was the only gene significantly associated with BIP-II (see Table S3.3).

**Figure 2:**
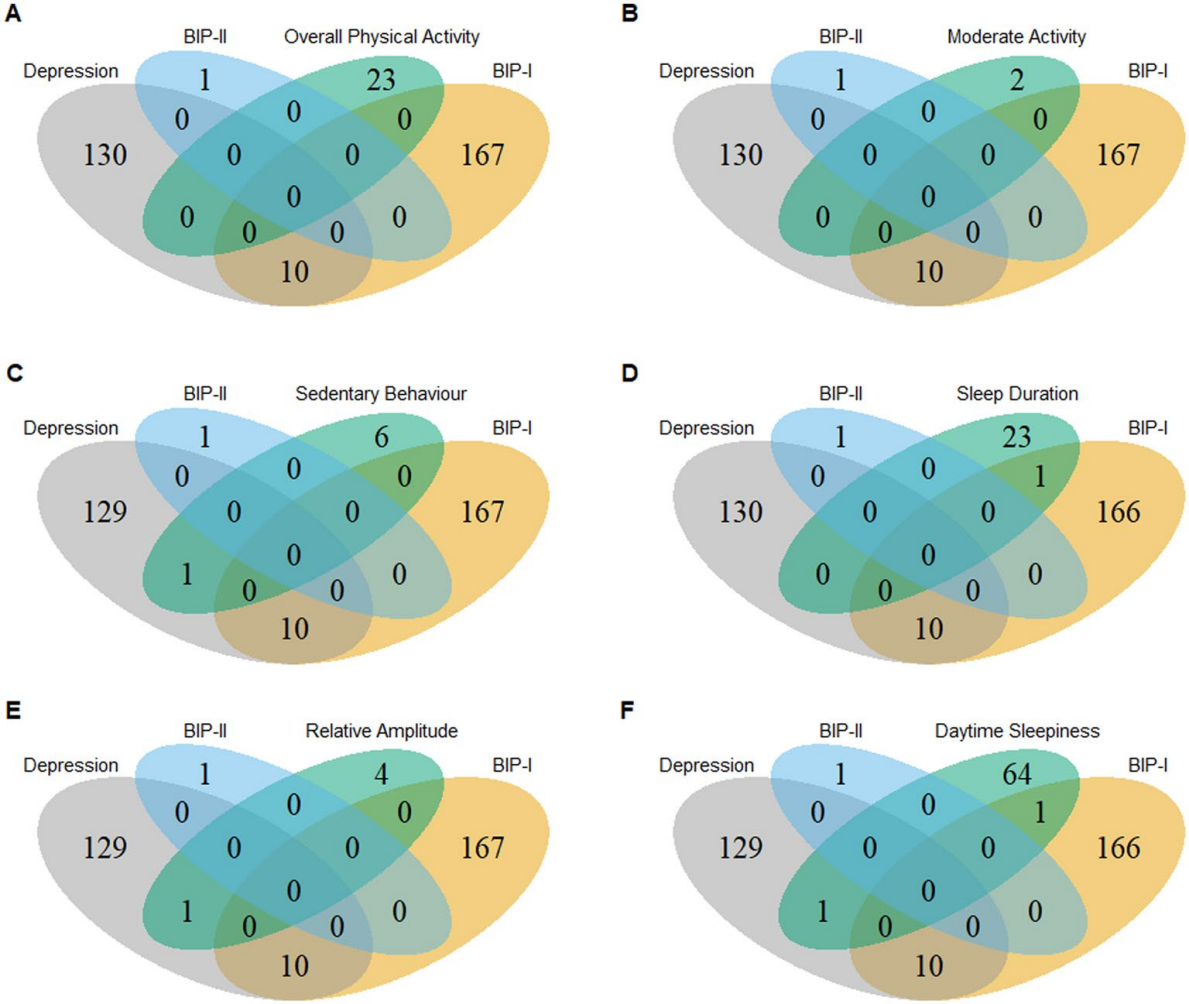
Venn Diagrams Showing Overlap of Genes Associated of Depression, BIP-I, BIP-II, with Biological Rhythms: (**A**) Overall Physical Activity, (**B**) Moderate Activity, (**C**) Sedentary Behaviour, (**D**) Sleep Duration, (**E**) Relative Amplitude, (**F**) Daytime Sleepiness. Gene-based associations were determined using MAGMA, and 17,861 genes available in all GWASs were tested. For the presented overlap, association was assigned based on a suggestive threshold *p* < 1 × 10^–5^.

The overlap of genes associated with mood disorders and biological rhythms (*p* < 1 × 10^–5^) is presented in Fig. 2. Depression showed an overlap with sedentary behaviour in the gene *MEF2C* (Z_dep_ = 4.53, p_dep_ = 2.95 × 10^–6^; Z_sb_ = 4.43, p_sb_ = 4.77 × 10^–6^, with relative amplitude in *CCDC36* (Z_dep_ = 4.30, p_dep_ = 8.66 × 10^–6^; Z_ra_ = 4.41, p_ra_ = 5.28 × 10^–6^), and with daytime sleepiness in *ERBB4* (Z_dep_ = 6.97, p_dep_ = 1.63 × 10^–12^; Z_ds_ = 4.39, p_ds_ = 5.80 × 10^–6^). BIP-I showed an overlap with sleep duration in *MSRA* (Z_bip-I_ = 5.69, p_bip-I_ = 6.26 × 10^–9^; Z_sd_ = 5.12, p_sd_ = 1.53 × 10^–7^) and daytime sleepiness in *CADM2* (Z_bip-I_ = 5.00, p_bip-I_ = 2.93 × 10^–7^; Z_ds_ = 5.90, p_ds_ = 1.83 × 10^–9^). BIP-II associated genes did not overlap with biological rhythms genes at the p-value threshold of *p* < 1 × 10^–5^.

In the targeted examination of circadian genes, significant associations (p_adj_ < 0.05) of depression with *NR1D1* (Z = 4.23, *p* = 1.16 × 10^–5^) and *PER1* (Z = 2.94, *p* = 0.002) were observed. BIP-I was associated with *ARNTL* (Z = 3.35, *p* = 0.0004), *CRY2* (Z = 3.15, *p* = 0.0008), and *RORB* (Z = 3.05, *p* = 0.001). BIP-II showed no significant association with any circadian gene. All circadian genes associated with mood disorders are presented in Supplementary Tables S4.1–S4.3.

## Discussion

In the present study, we used data from independent GWASs of mood disorders and biological rhythms and demonstrate that they have common genetic roots. Our results show that depression, BIP-I, BIP-II have commonalities but also distinct differences in their genetic correlations with biological rhythms. Notably, our study is the first to differentiate between the BIP subtypes BIP-I and BIP-II, revealing specific patterns of the two subtypes with biological rhythms. Our gene-level analysis shows that depression and BIP-I share genes with biological rhythms and that both mood disorders are associated with known circadian genes.

Epidemiological and clinical studies have repeatedly reported an inverse relation between higher levels of activity and depression^46,47^. Our findings now show that this inverse relationship has -at least partially- a common genetic basis. Our finding of a negative genetic correlation between increased physical activity (moderate activity) and depression is in line with the results from a recent twin study showing a genetic relationship between decreased depression and increased activity^26^, and also confirms an earlier study using GWAS data which reported negative genetic correlations of MD with overall physical activity as well as walking^36^.

BIP-I and BIP-II were positively genetically correlated with moderate activity and negatively with sedentary behaviour, implying that both BIP-I and BIP-II share genetic properties with increased physical activity. Our findings agree with previous findings showing a positive genetic correlation of BIP with moderate activity and walking^36^ based on an earlier BIP GWAS^48^. Placing the present findings in context, it should be noted that earlier works are based on BIP GWAS aggregating all subtypes in wherein the large majority of the patients were diagnosed with BIP-I^48^; the present analysis uses a larger dataset and separately examines BIP-II. Actigraphy studies suggest positive associations between physical activity and BIP during manic and hypomanic phases^9,10^; our results indicate that those relationships are partially due to shared genetic factors and not solely a result of the mood state in which they are observed.

Interestingly, our investigation of relative amplitude found negative genetic correlations with both depression and BIP-II, but not with BIP-I, which is a reflection of findings at the phenotypic level^13–16^. We note that the strongest and most statistically significant correlation in the present analysis was between depression and relative amplitude, suggesting this relationship as a target for further investigation and characterization. Physiological and molecular studies suggest that disruption of the molecular clock is strongly involved in mood symptoms observed in MD and BIP^30,49,50^; the present findings appear to suggest a connection on a wider genomic level. One study recently took such an approach using genome-wide data to investigate these relationships with polygenic risk scores (PRS)^40^. A PRS is a single score capturing the sum of an individual’s risk alleles weighted by GWAS summary statistics to quantify the genetic predisposition of an individual for a certain phenotype^34^. This study found that participants with an increased PRS for a disrupted circadian rhythm showed in turn an increased PRS for MD^40^. It should be noted that the measure relative amplitude, on which the summary statistics were based, is a composite index representing the difference between most and least active hours of the day and does not fully describe rest-activity cycles. More comprehensive measures will be needed to better investigate the links between circadian rhythmicity and mood disorders.

Symptoms such as insufficient sleep duration and frequent awakenings are often reported in mood disorders^51^. Genetic studies report a high genetic overlap of sleep-related phenotypes (e.g., insomnia, chronotype, sleep duration) with MD and BIP^52,53^. An earlier GWAS using self-report measures of sleep duration reported a significant genetic correlation with BIP, but not with depressive symptoms^54^; in the present study, significant genetic correlations of objectively measured sleep duration were not observed with MD or either BIP subtypes. This may be due to the difference in assessment methodology but may also be clarified when larger samples of objectively measured sleep duration become available. Here, we observed that depression, BIP-I, and BIP-II are significantly positively correlated with daytime sleepiness. With respect to MD, this confirms findings of an earlier study that found a genetic correlation between daytime sleepiness and depressive symptoms^35^. The same study examined BIP as well in a smaller sample but did not look at the subtypes separately^35^; the increased sample size and power in the present study enabled detection of significant correlations of daytime sleepiness in BIP-I and BIP-II, which is a novel finding.

When we compared the genetic correlations between mood disorders, the most prominent differences were found between depression and BIP-I, which showed genetic correlations with opposite directions for all objectively assessed traits besides relative amplitude, although in the same direction, correlation strength differed significantly. These differences may be causally linked to the clinical symptoms observed during the depressive episodes in MD (generally low activity) and during manic episodes (high activity) in BIP-I. BIP-II showed an intermediate correlational pattern, with the only significant differences in relative amplitude and daytime sleepiness being with BIP-I and a significant difference in moderate activity to depression. Notably, depression and BIP-II showed a closer resemblance in sleep duration, relative amplitude, and daytime sleepiness than BIP-I and BIP-II, which suggests a stronger link between genetics of depressed features with these phenotypes. At the same time, BIP-I and BIP-II are more similar to each other when compared to depression with respect to increased activity phenotypes. It is of interest that the strongest similarities between the mood disorders were observed in daytime sleepiness, suggesting that it shares common genetic etiology with all mood disorder types. In summary, the discovered similarities and differences appear to be clues to delineating these mood disorders with respect to each other.

The gene-based analysis revealed several genome-wide significant genes associated with depression (top-ranked: *HIST1H2BN*, *HIST1H3J*, *SORCS3*) and BIP-I (top-ranked: *CACNA1C*, *MAD1L1*, *PLEC*); and BIP-II was significantly  associated with *SLIT3*. These genes have been reported previously for the respective GWAS^37,38^.

Analysis of overlapping genes suggests potential pleiotropic targets for the investigation of mood disorders and biological rhythms. Depression and sedentary behaviour showed an overlap in *MEF2C*, a protein-coding gene involved in processes such as cell differentiation and neurogenesis; studies implicate *MEF2C* in neuropsychiatric disorders (e.g., schizophrenia and autism)^55^ and it is also involved in muscle activity and exercise^56^. *CCDC36*, the gene overlapping between depression and relative amplitude, is important for meiosis and other cellular processes; it has been reported to contribute to both MD and attention deficit and hyperactivity disorder^57^. The overlapping gene between depression and daytime sleepiness was *ERBB4*, part of the Tyr protein kinase family, was found to be associated with schizophrenia endophenotypes^58^. Also, a study investigating sleep regulation via the EGFR signalling pathway found genetic variants in *ERBB4* to be associated with excessive daytime sleepiness^59^. BIP-I and sleep duration were both associated with *MSRA*, which is engaged in protein repair processes; *MSRA* was associated with nine psychiatric disorders in a study investigating pleiotropic effects^60^; it has also been linked to neuroticism^61^ and sleep behaviour^62^. *CADM2*, which was overlapping in BIP-I and daytime sleepiness, belongs to the immunoglobulin superfamily and has been implicated in various genetic studies investigating psychological and physical traits, such as obesity^63^, cognitive function^64^, and physical activity^65^.

Findings from the targeted examination of clock genes revealed several significant associations with depression (*NR1D1* and *PER1*) and BIP-I (*ARNTL*, *CRY2*, *RORB*) underlining the close relationship with mood disorders. *NR1D1* regulates the expression of core clock genes and is involved in metabolic and immunological processes; it has been reported to be associated with both depression^66^ and bipolar disorder^67^. *PER1* is involved in the transcription and translation of core clock components and is involved in a wide range of physiological functions (e.g.metabolism, sleep, and the endocrine and immune systems); changes in *PER1* expression have been found in patients with depression compared to healthy controls^67^. *ARNTL* acts as an activator of transcriptional processes in the circadian clock machinery and is known as a metabolic modulator^68^; it has been implicated in affective disorders^67^. *CRY2*, a repressor of transcription in the circadian clock machinery, has been shown to be related to depression^69^, and bipolar disorder^70^. The nuclear receptor *RORB* has been shown to be associated with bipolar disorder^71^. Even though circadian genes did not reach genome-wide significance, they support findings from previous studies indicating a close genetic relationship between mood disorders and circadian rhythms on a genome-wide gene level.

The present study had certain limitations. We used the largest currently available cohorts for the respective phenotypes; however, sample sizes and statistical power of the GWASs differed. In particular, BIP-II and relative amplitude samples were smaller than for the other GWASs. All GWASs included exceed the SNP heritability Z-score threshold of 4 which is recommended for genetic correlation analyses (see Tables S1.1–S1.3) and we could expect more significant findings with larger samples. Another limitation that bears mentioning is that it cannot be excluded that some of the individuals in the biological rhythms dataset were suffering from a mood disorder (lifetime or acute)^72^. However, in the light of the relatively low lifetime frequency of BIP and the decreased likelihood that individuals with MD or BIP have participated in the accelerometer recording during an acute mood episode, it is unlikely that the genetic correlation between biological rhythms and mood disorders could result from the biorhythms observed in these individuals at the time of assessment.

These results show that clinically observed relationships of mood disorders and biological rhythms have a common genetic basis, and indicate that alterations in biological rhythms observed in mood disorder patients are linked to the genetic vulnerability for the specific disorder, and not only to the current mood episode. Biological rhythms should be given more attention in the study of mood disorders and require greater consideration with respect to assessment and treatment. Links between mood disorders and genetic components underlying other physiological traits also under circadian control (e.g., hormonal secretion, body temperature) should be investigated to enhance our understanding of this complex relationship. As sample sizes of GWASs increase, overlapping genes and pathways can be identified with more certainty, enabling investigation of the causality of the observed genetic relationship between mood disorders and biological rhythms. To further extend these findings, future studies in the field could investigate this relationship also on the individual level, assessing mood disorders and biological rhythm phenotypes with more refined approaches such as ambulatory assessment, while at the same time incorporating genetic data. Furthermore, incorporating epigenetic and gene expression assessments into research designs will allow exploration of underlying mechanisms, in particular those that change over time. Implementation of these multimodal assessments will not only give deeper insight into shared mechanisms but also shed light on how they interact with the environment. If shared genetic factors jointly affect biological rhythms and liability to mood disorders, further investigation may allow targeting of these factors in treatment, providing potential avenues of improvement for therapeutic approaches.

## Supplementary Information

Supplementary Information.

## Data Availability

Summary statistics for depression were downloaded from the Downloads section on the Psychiatric Genomics Consortium Webpage (https://www.med.unc.edu/pgc/download-results/, March 19th, 2020)^37^. BIP-I and BIP-II summary statistics were obtained from the authors of the original GWAS (November, 2020)^38^. The summary statistics for overall physical activity, moderate activity, sedentary behaviour, and sleep duration were downloaded via the following link: https://ora.ox.ac.uk/objects/uuid:ff479f44-bf35-48b9-9e67-e690a2937b22 (retrieved March 13th, 2020)^39^. Relative amplitude summary statistics were retrieved from http://researchdata.gla.ac.uk/928/ (January 1st, 2020) and for daytime sleepiness from the Sleep Disorder Knowledge Portal (SDKP) website http://www.sleepdisordergenetics.org (June 9th, 2020)^40,35^.

## Code availability

Code used for this analysis can be found here: https://github.com/bulik/ldsc/wiki/Heritability-and-Genetic-Correlation (retrieved February 4th, 2022) and https://ctg.cncr.nl/software/magma (retrieved July 16th, 2022) or can be made available by the corresponding author based on reasonable request.
